# Supplementary material for: Measurement of airborne particle emission during surgical and percutaneous dilatational tracheostomy COVID-19 adapted procedures in a swine model: Experimental report and review of literature
Source: PLoS One. 2022 Nov 23;17(11):e0278089. doi: 10.1371/journal.pone.0278089 (PMC9683587; doi:10.1371/journal.pone.0278089)
Supplement: S1 Fig — All data from percutaneous dilatational (PDT) and surgical tracheostomy (ST) procedures are shown. Mean baseline variation depends on size particles. For 0.5 and 1 μm particles, normal baseline variations were in 10% range. For 3 μm particles, normal baseline variations were in 25% range. PDT-0 and ST-0 were preliminary measures performed without acute respiratory distress syndrome (ARDS) induction. The level of particles emitted was 1–20 times higher using ARDS, which justified the systematic induction of ARDS. ST-6 and PDT-6 were excluded because of important baseline variations due to a slightly ajar window in the experiment room. (PDF) [file pone.0278089.s001.pdf]

Potential Confounders ; preliminary tests

(% of variation of the baseline)

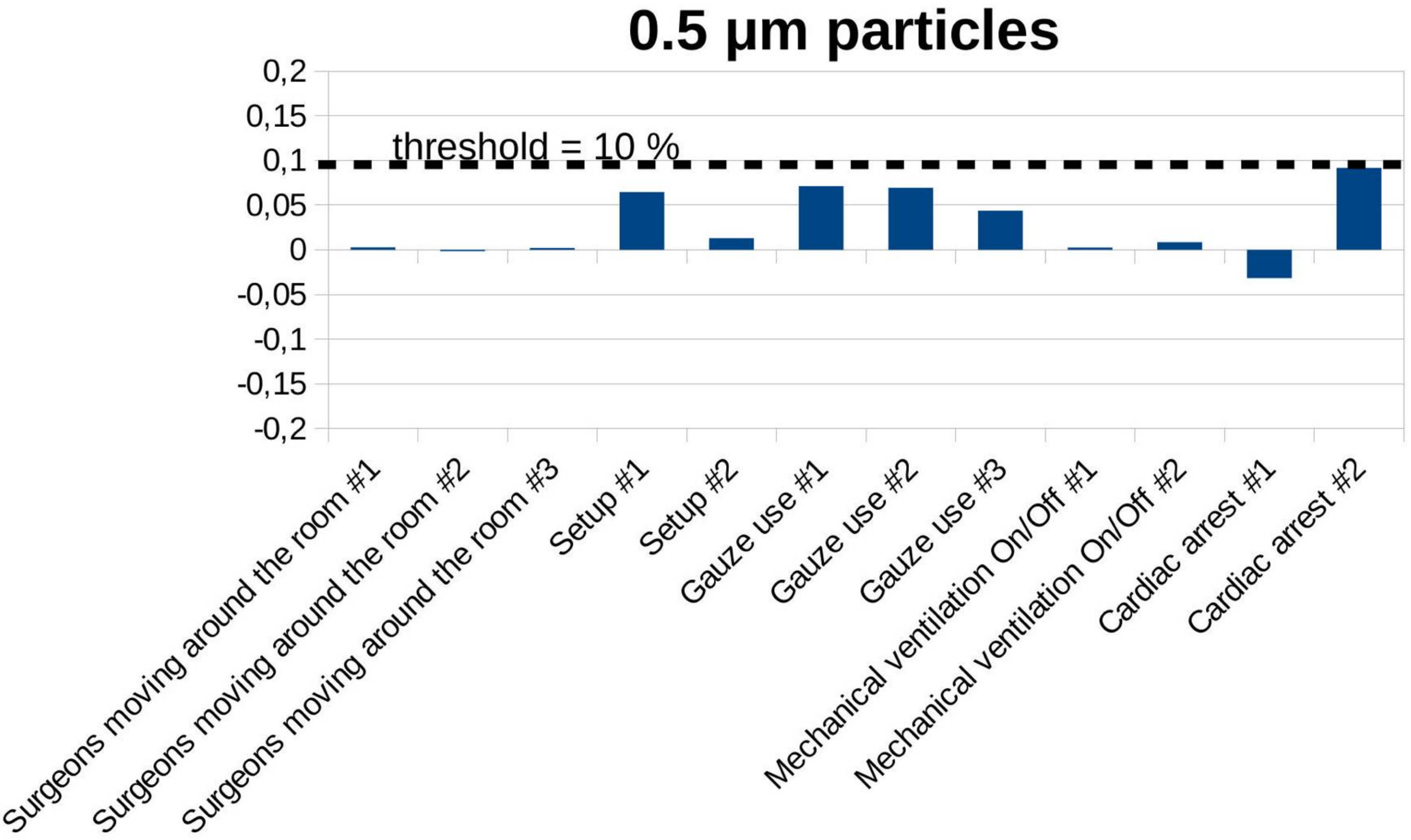

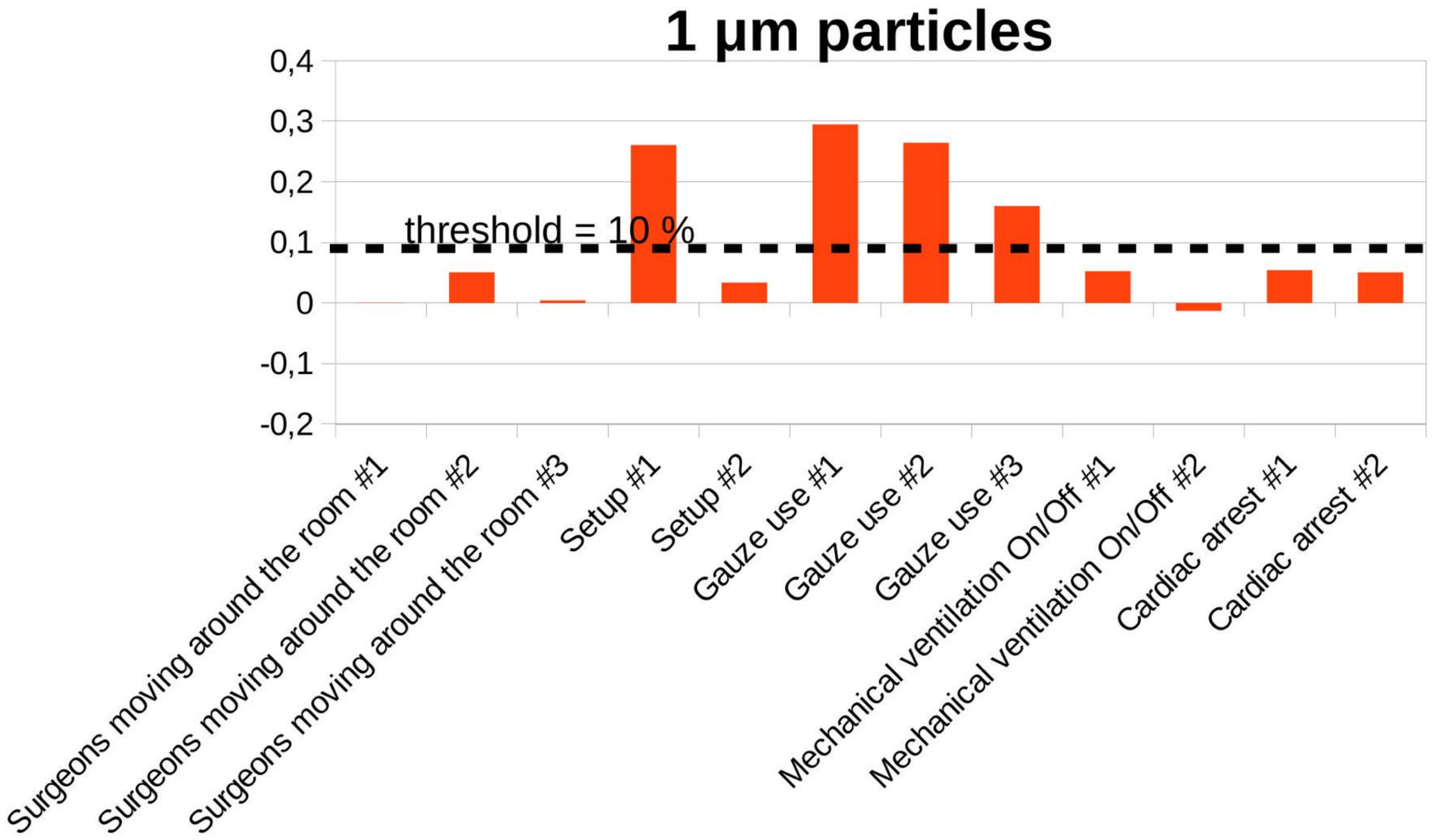
1 µm particles
0,4
0,3
0,2
0,1
0
-0,1
-0,2
threshold = 10 %
Surgeons moving around the room #1
Surgeons moving around the room #2
Surgeons moving around the room #3
Setup #1
Setup #2
Gauze use #1
Gauze use #2
Gauze use #3
Mechanical ventilation On/Off #1
Mechanical ventilation On/Off #2
Cardiac arrest #1
Cardiac arrest #2

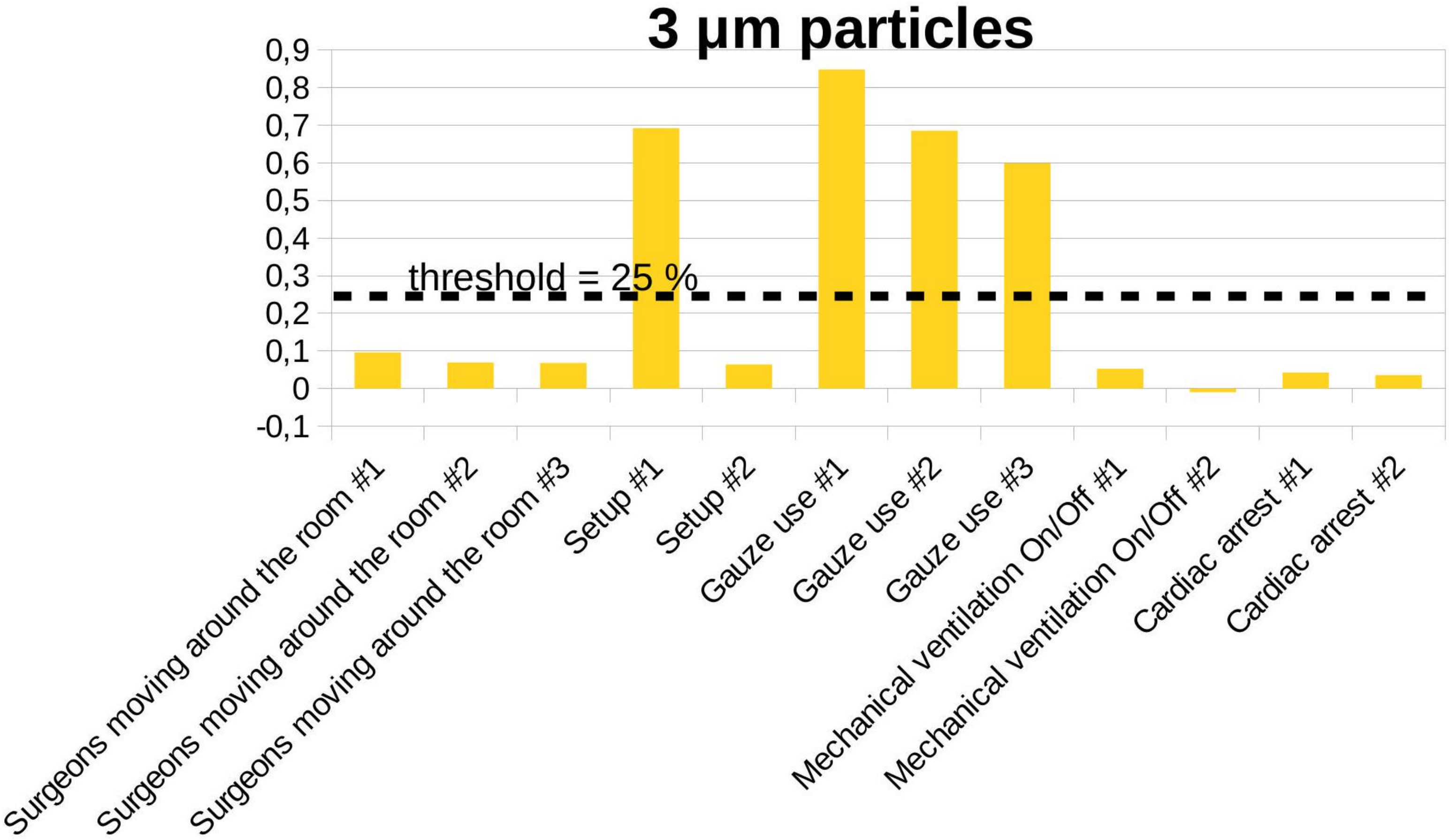

Supplementary Figure 1

Normal variations of the baseline and induced by provoked leaks. All data from percutaneous dilatational (PDT) and surgical tracheostomy (ST) procedures are shown. Mean baseline variation depends on size particles. For 0.5 and 1 µm particles, normal baseline variations were in 10% range. For 3 µm particles, normal baseline variations were in 25 % range. PDT-0 and ST-0 were preliminary measures performed without acute respiratory distress syndrome (ARDS) induction. The level of particles emitted was 1-20 times higher using ARDS, which justified the systematic induction of ARDS. ST-6 and PDT-6 were excluded because of important baseline variations due to a slightly ajar window in the experiment room
