# Supplementary material for: Measurement of airborne particle emission during surgical and percutaneous dilatational tracheostomy COVID-19 adapted procedures in a swine model: Experimental report and review of literature
Source: PLoS One. 2022 Nov 23;17(11):e0278089. doi: 10.1371/journal.pone.0278089 (PMC9683587; doi:10.1371/journal.pone.0278089)
Supplement: S2 Fig — Hatching in the background: Intensive care ventilator on; white in the background: Intensive care ventilator off; * significant peaks related to a breach in ventilation circuit; † significant peaks related to an artifact (like dry gauze use). Baseline, procedure and intentional aerosol-generating maneuver (control) are shown. (PDF) [file pone.0278089.s002.pdf]

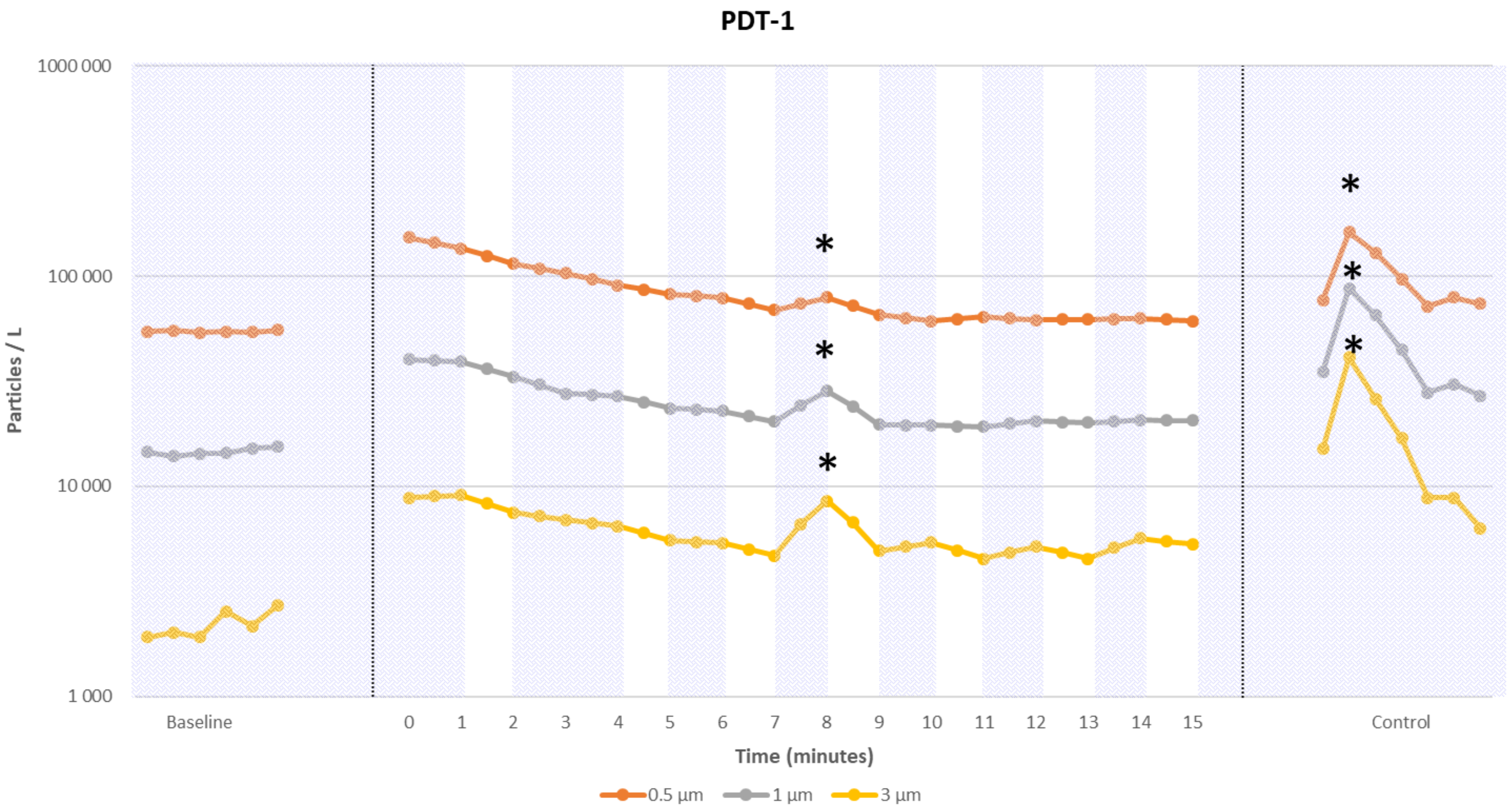
PDT-1
1000 000
100 000
10 000
1 000
Particles / L
Baseline
0
1
2
3
4
5
6
7
8
9
10
11
12
13
14
15
Control
Time (minutes)
*
*
*
*
*
*
0.5 µm
1 µm
3 µm

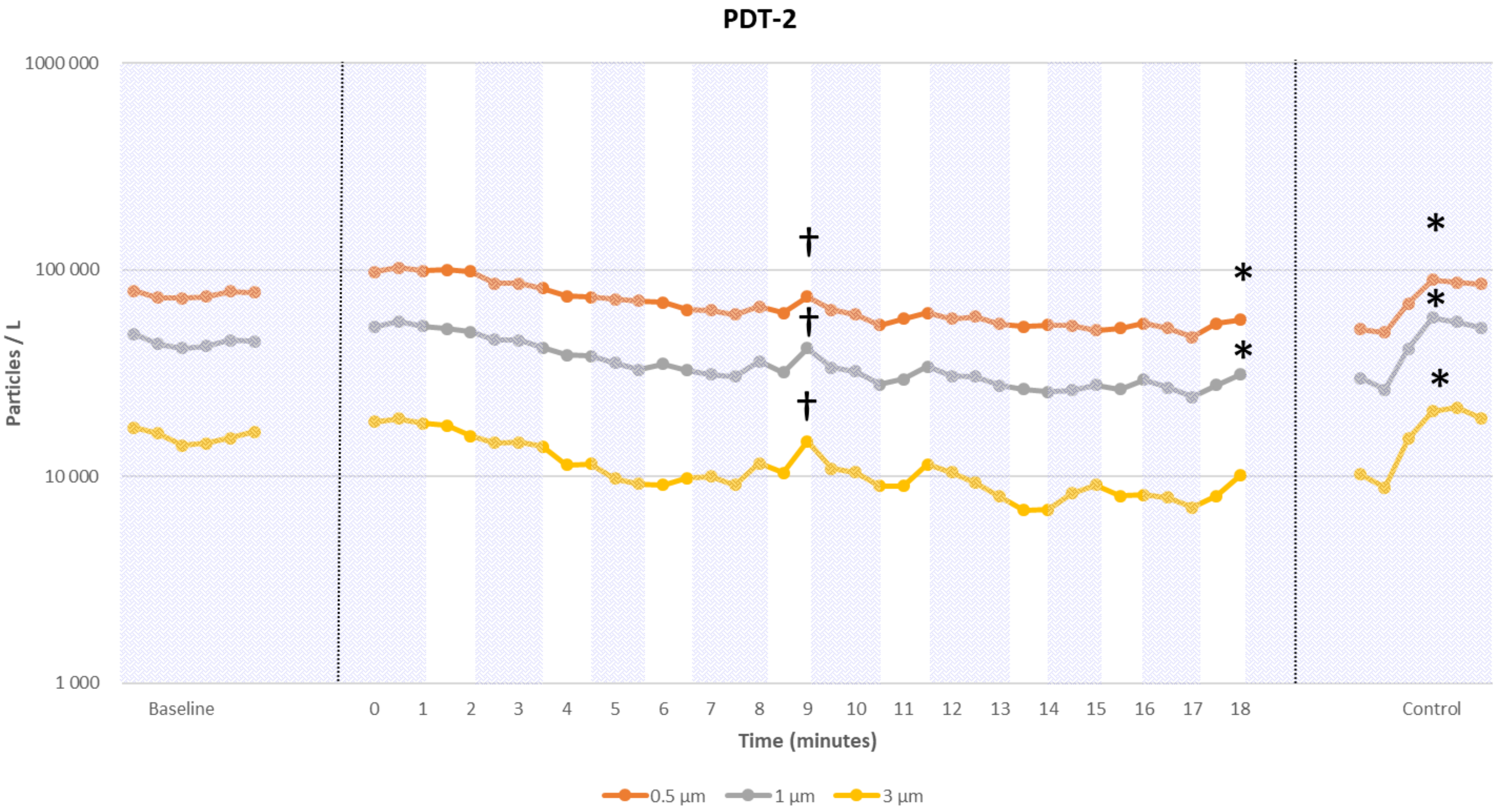
PDT-2
1000 000
100 000
10 000
1 000
Particles / L
Baseline
0
1
2
3
4
5
6
7
8
9
10
11
12
13
14
15
16
17
18
Control
Time (minutes)
†
†
†
*
*
*
*
*
*
0.5 µm
1 µm
3 µm

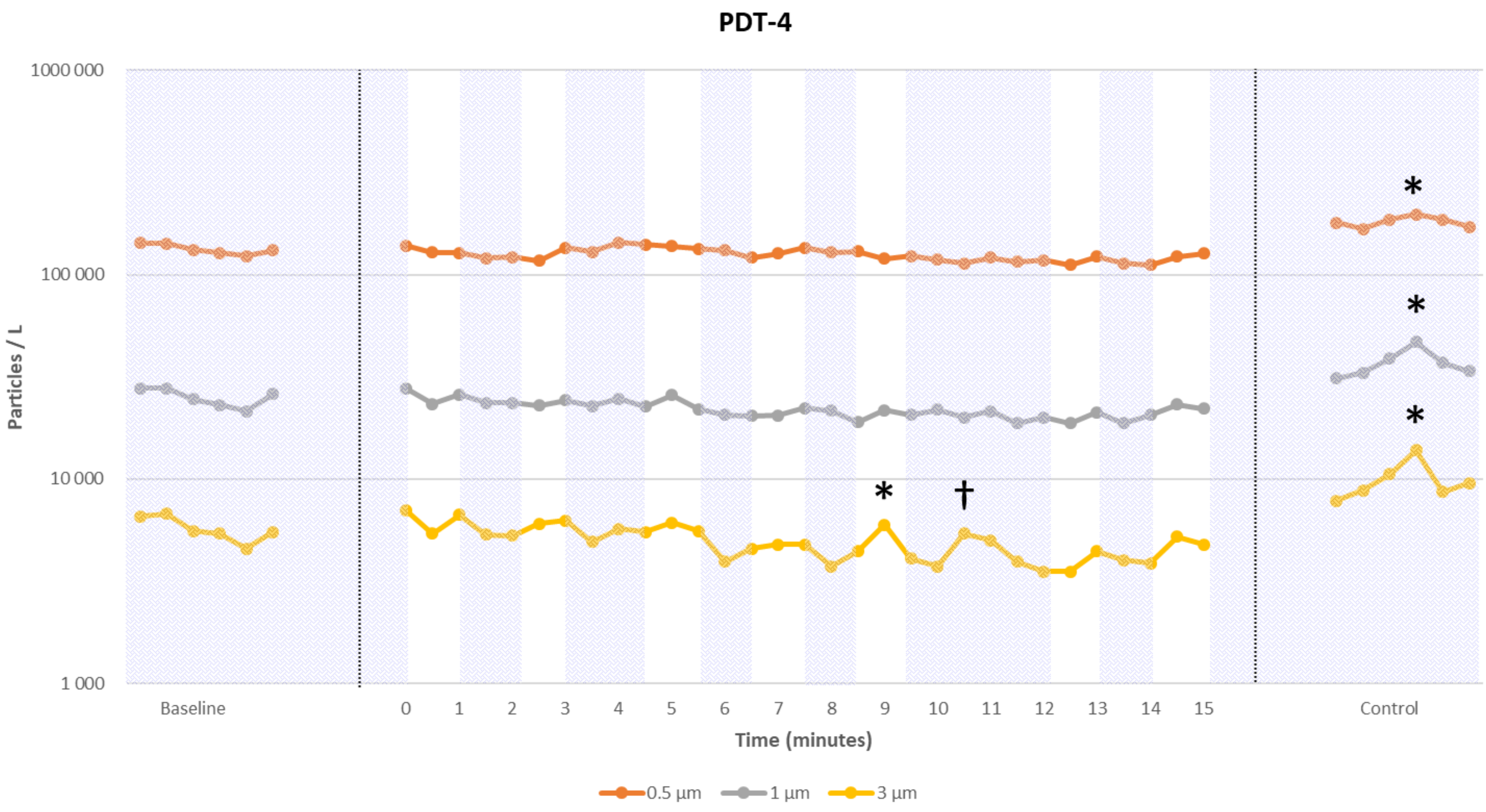
PDT-4
1000 000
100 000
10 000
1 000
Particles / L
*
*
*
*
†
Baseline
0
1
2
3
4
5
6
7
8
9
10
11
12
13
14
15
Control
Time (minutes)
0.5 µm
1 µm
3 µm

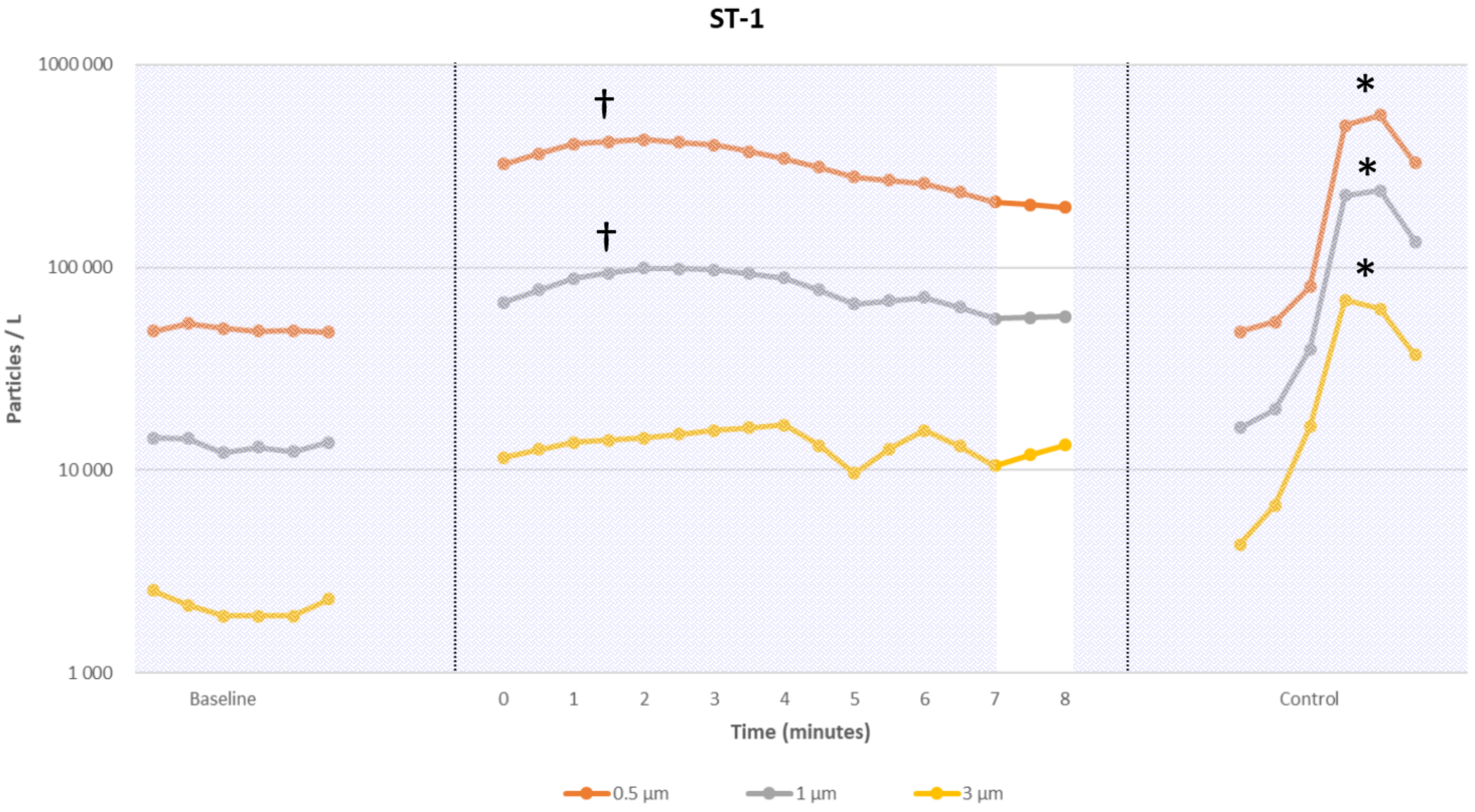
ST-1
1000 000
100 000
10 000
1 000
Particles / L
†
†
*
*
*
Baseline
0
1
2
3
4
5
6
7
8
Control
Time (minutes)
0.5 µm
1 µm
3 µm

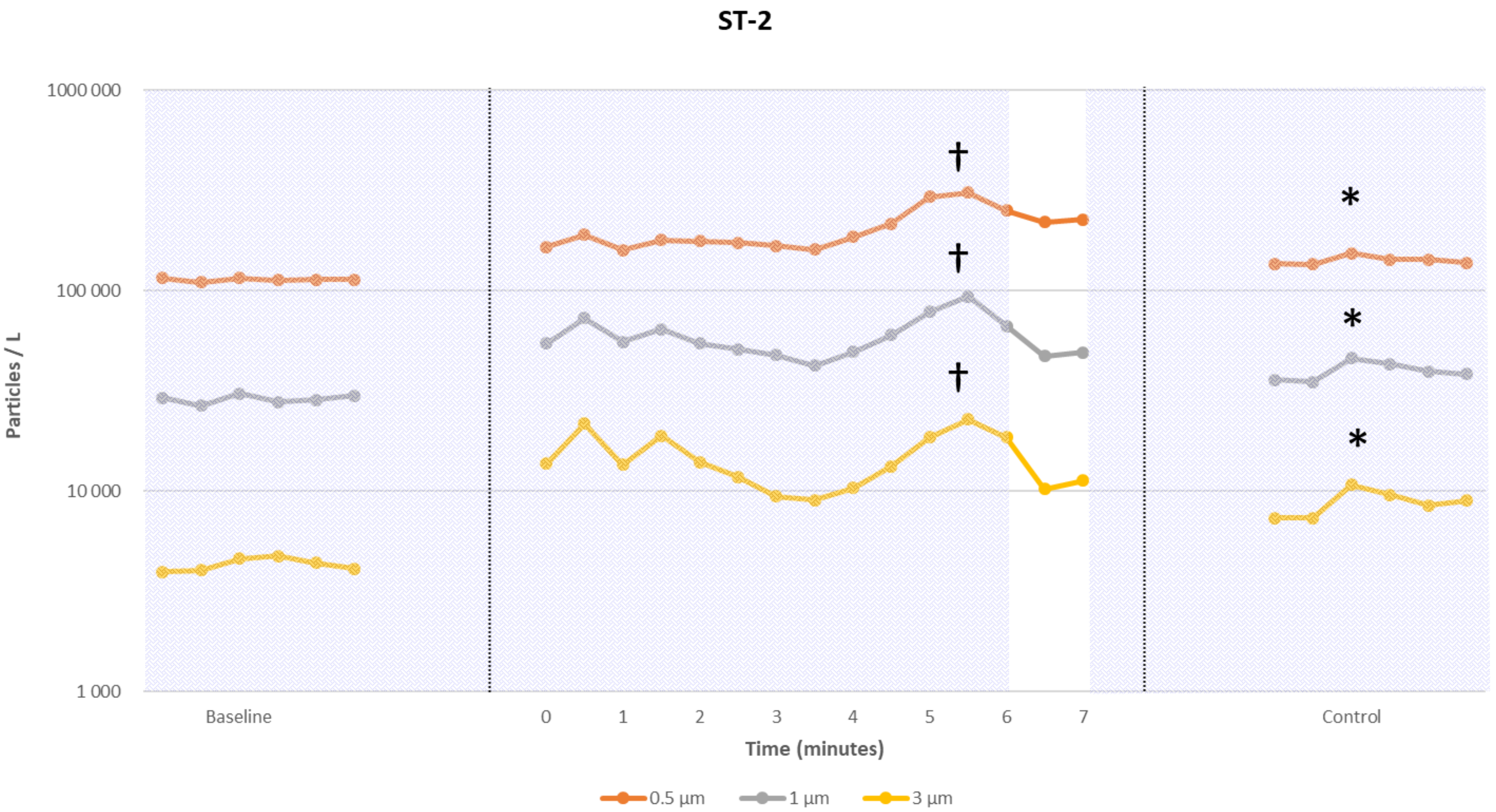
ST-2
1000 000
100 000
10 000
1 000
Particles / L
Baseline
0
1
2
3
4
5
6
7
Control
Time (minutes)
0.5 µm
1 µm
3 µm
†
*

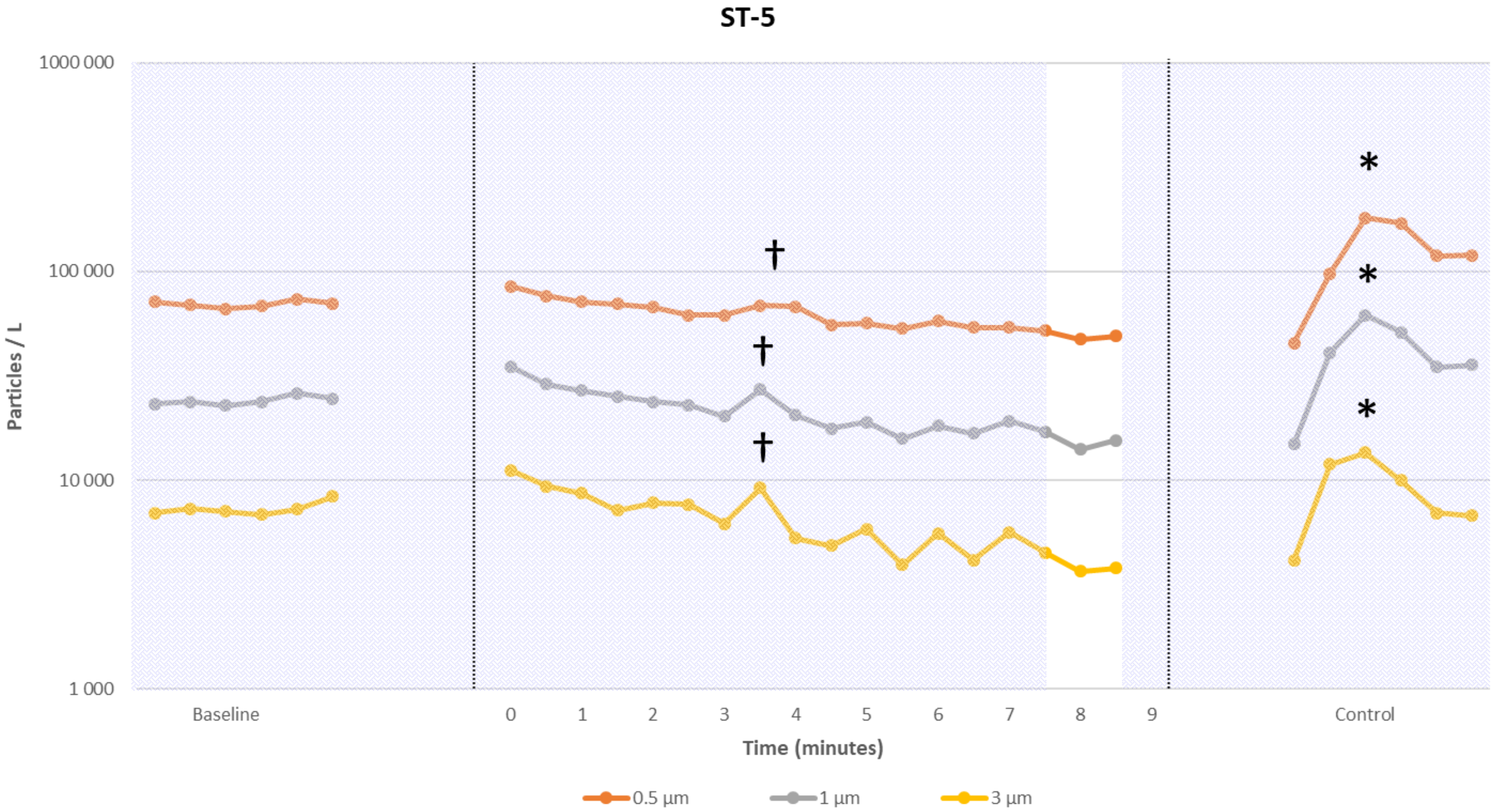
ST-5
1000 000
100 000
10 000
1 000
Particles / L
Baseline
0
1
2
3
4
5
6
7
8
9
Control
Time (minutes)
0.5 µm
1 µm
3 µm
†
*

**Supplementary Figure 2**

Particle count (logarithmic scale) during remaining percutaneous dilatational (PDT) and surgical tracheostomy (ST) procedures. Hatching in the background: intensive care ventilator on; white in the background: intensive care ventilator off; * significant peaks related to a breach in ventilation circuit; † significant peaks related to an artifact (like dry gauze use). Baseline, procedure and intentional aerosol-generating maneuver (control) are shown.
