# Supplementary figures and images for: Measurement of airborne particle emission during surgical and percutaneous dilatational tracheostomy COVID-19 adapted procedures in a swine model: Experimental report and review of literature
Source: PLoS One. 2022 Nov 23;17(11):e0278089. doi: 10.1371/journal.pone.0278089 (PMC9683587; doi:10.1371/journal.pone.0278089)

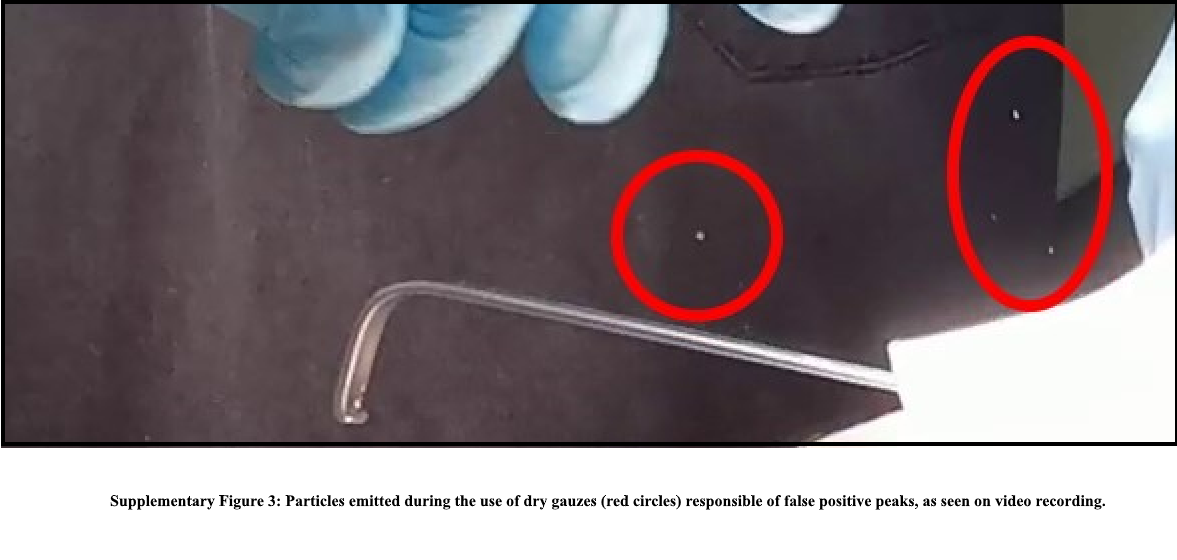

Supplement: S3 Fig — (TIF) [file pone.0278089.s003.tif]
